# Ocularultrasound versus SD-OCT in early papilledema differentiation

**DOI:** 10.6026/973206300221905

**Published:** 2026-03-31

**Authors:** Aditi Mishra, Sankriti Ukey, Ankita Baghel, Eva Rani Tirkey, Sachin Parmar

**Affiliations:** 1 Department of Ophthalmology, Nand Kumar Singh Chauhan Medical College, Khandwa, Madhya Pradesh, India; 2 Department of Ophthalmology, Shyam Shah Medical College, Rewa, Madhya Pradesh, India; 3 Department of Community Medicine Department V.K.S. Government Medical College, Neemuch, Madhya Pradesh, India

**Keywords:** Papilledema, optic disc drusen, tomography, optical coherence

## Abstract

Differentiating early papilledema from pseudopapilledema remains a considerable diagnostic challenge in neuro-ophthalmology due the risks surrounding unnecessary neuroimaging or under-treatment of elevated ICP. Therefore, it is of interest to study of ocular ultrasonography and SD-OCT was carried out in 60 patients with elevation of the optic disc from September 2022 to February 2024. Papilledema was defined as optic nerve sheath diameter >3.3 mm, a positive 30°C test and crescent sign while pseudopapilledema was often associated with hidden disc drusen. SD-OCT had higher sensitivity than ultrasonographic examination (86.7% vs 81.7%) and eyes with papilledema showed thicker peripapillary RNFL, increased ONH volume, decreased GCIPL, enlargement of the blind spot or reduced contrast sensitivity and colour desaturation. The two modalities complement each other - SD-OCT provides quantification and allows differentiation whereas ultrasonography can show anatomical biomarkers, together allowing the early detection of intracranial hypertension as well as avoiding invasive investigations in cases of pseudopapilledema.

## Background:

Optic disc elevation represents a diagnostic challenge in neuro-ophthalmology clinics and necessitates accurate discrimination between true papilledema and pseudopapilledema. Papilledema is defined as optic disc swelling resulting from raised intracranial pressure and mandates prompt identification to avert irreversible visual loss or address life-threatening causes such as intracranial mass lesions or venous sinus thrombosis [1]. Pseudopapilledema, in contrast, refers to optic nerve head (ONH) elevation due to anomalous anatomical variants, most commonly optic disc drusen or congenital anomalies and does not pose the same risk profile or require urgent intervention. Clinical distinction between these two conditions is often complicated by overlapping fundus features such as blurred disc margins, elevation, or absence of physiological cupping [2]. Traditional evaluation of optic disc elevation relies on a combination of comprehensive clinical examination, fundus photography and ancillary tests. Standard visual function assessments, including perimetry and visual acuity measurement, generally lack specificity in differentiating mild papilledema from pseudopapilledema [3]. Fundoscopic findings such as venous pulsations, presence of disc hemorrhages, or macular exudates can aid in diagnosis but may not be definitive, particularly in cases of early or low-grade disc edema. Consequently, noninvasive, objective imaging modalities have been increasingly utilized to enhance diagnostic accuracy in cases of optic disc elevation [4, 5]. Ocular ultrasonography offers real-time structural assessment of the optic nerve sheath and retrobulbar space. The optic nerve sheath diameter (ONSD), measured using B-scan ultrasonography, has emerged as a clinically useful parameter; an ONSD of ≥3.3 mm, especially with a positive 30-degree test (demonstrating sheath compliance), strongly suggests papilledema [6]. Sensitivity of this measure in distinguishing papilledema from pseudopapilledema has been consistently high, with reported values up to 90%, although specificity may be somewhat lower due to overlap with some anatomic variants [7]. Ultrasonographic signs such as the "crescent sign" and the absence of visible buried optic disc drusen further improve diagnostic yield in differentiating true from false disc edema [8]. Spectral-domain optical coherence tomography (SD-OCT) enables high-resolution cross-sectional imaging of the retinal nerve fiber layer (RNFL) and ONH architecture. Peripapillary RNFL thickening is a characteristic of papilledema, reflecting axoplasmic flow stasis at the ONH, while in pseudopapilledema, RNFL thickening is generally mild or absent. SD-OCT also quantifies optic nerve head volume (ONHV), Bruch's membrane opening (BMO) and ganglion cell-inner plexiform layer (GCIPL) thickness, parameters that show distinctive profiles between papilledema and congenital disc anomalies. Longitudinal or baseline changes in pRNFL, ONHV and BMO have demonstrated high discriminatory power, particularly in mild optic disc elevation (Frisen grades I and II). OCT can also directly visualize optic disc drusen as hyper reflective deposits, aiding in identification of pseudopapilledema [6]. Optic disc swelling may occur because of many etiologies, including true or pseudopapilledema. Determining effective and accurate differential diagnostic criteria in these cases will prevent unnecessary examinations and procedures during the diagnosis [9]. Head-to-head studies comparing ocular ultrasonography and SD-OCT highlight complementary roles for these modalities in clinical practice. OCT provides superior spatial resolution for quantitative retinal layer analysis, while ultrasonography excels in detecting retrobulbar nerve and sheath alterations. The integration of clinical assessment, ocular ultrasonography and SD-OCT markedly enhances the sensitivity and specificity of diagnosing papilledema, optimally guiding further investigations such as magnetic resonance imaging and neuroimaging reserved for ambiguous or high-risk presentations [10]. Therefore, it is of interest to assess ocular ultrasonography and SD-OCT as complementary, noninvasive modalities for accurately differentiating Grade I-II papilledema from pseudopapilledema, to facilitate timely neuroimaging and management of suspected raised intracranial pressure while avoiding unnecessary invasive procedures.

## Materials and Methods:

The current cross-sectional observational study titled Ocular Ultrasonography and Spectral Domain Optical Coherence Tomography to Differentiate Grade I and II Papilledema vs Pseudopapilledema in Clinically Diagnosed Optic Disc Elevation was done in the month of September 2022 to February 2024 at the Neuro-ophthalmology Clinic, Department of Ophthalmology, Shyam Shah Medical College, Rewa. The aim was to make a comparison between the diagnostic sensitivity of ocular ultrasonography and SD-OCT parameters in the distinction of early- grade papilledema versus pseudopapilledema. Sixty patients who fit the selection criteria were enrolled.

## Study design:

Observational study which is cross sectional.

## Selection of cases:

Those patients who had optic disc elevation in fundus examination were included. According to the Modified Frisen Grading System.

## The cases were divided into following categories:

[1] Grade I papilledema - sensitive C-shaped disc edema halo with intact temporal margin.

[2] Grade II papilledema - peripheral halo of edema that is indicative of low grade papilledema.

[3] Pseudo papilledema - druse of optic disc, tilted optic disc, optic disc hypoplasia.

## Inclusion criteria:

[1] Clinical diagnosis of Grade I and II papilledema.

[2] Cases with pseudo papilledema presentation of fundus examination.

## Exclusion criteria:

[1] Age below 18 years.

[2] Hypertensive or diabetic retinopathy.

[3] Toxic/trumbo optic neuropathies.

[4] Grade > II Papilledema on Modified Frisen Grading.

## Data collection:

All the participants provided informed consent in writing upon being informed about the aim of the study. An elaborate demographic history, medical history and ophthalmic symptoms were noted. All patients were thoroughly systemically and ocularly assessed.

## Ophthalmic evaluation:

Standardized ophthalmic examination was done on all subjects enrolled and this included: Snellen chart Best Corrected Visual Acuity (BCVA), Intraocular Pressure (IOP) by applanation tonometer of Goldman. Slit-lamp biomicroscopy of anterior part, lens and vitreous. Fundus examination and after pharmacologic dilation Fundus examination and photography with slit-lamp using Direct and indirect ophthalmoscopy with Volk 90D lens. The Modified Frisen Grading System was used in recording and grading disc margin, color, cupping and vessel pattern, as well as optic disc elevation. A Topcon TRC-50DX fundus camera was used to take fundus photographs. Additional Investigations all participants underwent: Perimetry (Humphrey Field Analyzer), color vision, contrast sensitivity, Amsler grid and color desaturation, Optic nerve head ocular ultrasound, RNFL and optic nerve head Spectral Domain Optical Coherence Tomography (SD-OCT), MRI brain and orbit MR venography to rule out intracranial etiology, Lumbar puncture accompanied by measurement of the underlying pressure in the CSF and a biochemical analysis where necessary.

## Results:

The current study included 60 patients, divided into two groups of 30 patients each: one for papilledema and one for pseudopapilledema. The average age of patients with papilledema was 35.33 ± 12.82 years, while that of the pseudopapilledema group was 40.67 ± 13.16 years; females were the majority in both groups. The papilledema group had a mean BMI of 28.2 ± 4.9, which was much higher than the pseudopapilledema group's mean BMI of 23.2 ± 4.5 (p < 0.01). This group also had a higher percentage of overweight and obese people. The majority of patients were aged 18 to 30 years, with a smaller percentage of cases occurring in individuals over 60 years (Table 1 - see PDF). Clinically, headache was the most prevalent presenting symptom in papilledema (80%), succeeded by asthenopia, transient visual obscuration and nausea. Most people in both groups still had good vision, but the papilledema group had slightly better average logMAR scores. Patients with papilledema had much higher intraocular pressure (16.3 ± 2.9 mmHg) than those with pseudopapilledema (14.9 ± 1.5 mmHg, p = 0.0012) (Table 2 - see PDF). Ocular ultrasonography and SD-OCT results exhibited unique patterns across groups. The average optic nerve sheath diameter (ONSD) was significantly greater in cases of papilledema (mean 4.6 ± 0.6 mm) than in those of pseudopapilledema (mean 3.1 ± 0.5 mm, p < 0.001). In papilledema, the RNFL thickness and optic nerve head volume on SD-OCT were significantly higher, whereas the GCIPL measurements were lower. The crescent sign and positive 30°C test were helpful in telling the two conditions apart (Table 3 - see PDF). An evaluation of functional vision indicated that 40% of eyes with papilledema exhibited an enlargement of the blind spot, whereas no such enlargement was observed in eyes with pseudopapilledema. Contrast sensitivity and colour desaturation abnormalities were more evident in cases of papilledema (Table 4 - see PDF). In terms of diagnostic effectiveness, SD-OCT had a higher sensitivity for finding papilledema (86.7%) than ultrasonography (81.7%). However, both tests had similar specificity and predictive values. Neuroimaging results corroborated clinical diagnoses in most cases, with space-occupying lesions, elevated intracranial pressure and venous sinus abnormalities commonly observed in papilledema (Table 5 - see PDF).

The average age of patients with papilledema was 35.33 ± 12.82 years, while that of the pseudopapilledema group was 40.67 ± 13.16 years; females were the majority in both groups which is corresponds with the finding of Sekhri *et al.* [11] and Rosa *et al.* [12] in their study also preponderance of female gender is present. While the study done by Carta *et al.* [13] shown contrast with this study where gender distribution is equal. The observed greater retinal nerve fiber layer (RNFL) thickness and optic nerve head volume in papilledema compared to pseudopapilledema are consistent with the results reported by Bassi and Mohana [10] who demonstrated significantly thicker peripapillary RNFL in all quadrants in papilledema patients except the temporal quadrant, while pseudopapilledema cases exhibited localized thickening or normal RNFL values. Similar observations were reported by Fard *et al.* [14] who described characteristic hyper reflective sub retinal masses representing optic disc drusen in pseudo papilledema, aiding in distinction from true disc edema. The higher optic nerve sheath diameter (ONSD) measured on ocular ultrasonography in papilledema compared to pseudopapilledema in the present study (4.6 ± 0.6 mm vs. 3.1 ± 0.5 mm) corroborates previous studies, such as one by Saenz *et al.* [7] which demonstrated mean ONSD of 5.4 ± 0.6 mm in papilledema and 4.0 ± 0.3 mm in pseudopapilledema (p < 0.0001). These structural parameters strongly support the diagnostic usefulness of ultrasonography for detecting raised intracranial pressure. The higher mean BMI and predominance of overweight or obese individuals in the papilledema group further support the role of obesity as a predisposing factor for idiopathic intracranial hypertension (IIH). Szewka *et al.* [15] found a strong correlation between elevated BMI and severity of papilledema, where each 10-unit increase in BMI increased the odds of severe visual loss by 1.4-fold. Similarly, weight reduction has been associated with improved visual outcomes in IIH patients, further confirming the pathophysiological link between obesity and raised intracranial pressure. Functionally, headache, transient visual obscuration and enlarged blind spots were more common in papilledema, consistent with findings of previous series describing these symptoms as characteristic clinical features of raised intracranial pressure. In terms of diagnostic yield, SD-OCT showed higher sensitivity compared to ultrasonography in detecting papilledema, similar to the findings of Sibony *et al.* [16] who emphasized that quantitative parameters such as RNFL and GCIPL measurements on SD-OCT provide high diagnostic accuracy and are valuable for differentiating true edema from pseudopapilledema. These observations affirm the instrumental advantage of SD-OCT as a first-line tool in the evaluation of optic disc elevation, supported by concurrent ultrasonographic and neuroimaging assessments for definitive diagnosis.

## Conclusion:

Ocular ultrasonography with SD-OCT is effective in differentiating Grade I-II papilledema from pseudopapilledema based on structural biomarkers such as ONSD, pRNFL, ONH volume and GCIPL. The crescent sign and a positive 30°C test are key indicators of papilledema, whereas optic disc drusen are indicative of pseudopapilledema. SD-OCT was slightly more sensitive than ultrasonography, supporting its role as a primary modality complemented by ultrasound and neuroimaging. Early differentiation helps ensure timely management of elevated intracranial pressure and avoids unwarranted invasive procedures in pseudopapilledema.
